# Relationship between 25 Hydroxyvitamin D, Overweight/Obesity Status, Pro-Inflammatory and Oxidative Stress Markers in Patients with Type 2 Diabetes: A Simplified Empirical Path Model

**DOI:** 10.3390/nu13082889

**Published:** 2021-08-22

**Authors:** Adriana Florinela Cătoi, Mihaela Iancu, Alina Elena Pârvu, Andra Diana Cecan, Cristina Bidian, Elisabeta Ioana Chera, Ioana Delia Pop, Adrian Maximilian Macri

**Affiliations:** 1 Department of Pathophysiology, Faculty of Medicine, “Iuliu Hațieganu” University of Medicine and Pharmacy, 400012 Cluj-Napoca, Romania or florinela12@yahoo.com (A.F.C.); parvualinaelena@umfcluj.ro (A.E.P.); Andra.Cecan@umfcluj.ro (A.D.C.); Chera.Elisabeta@umfcluj.ro (E.I.C.); 2 Department of Medical Informatics and Biostatistics, Faculty of Medicine, “Iuliu Hațieganu” University of Medicine and Pharmacy, 400012 Cluj-Napoca, Romania; 3 Department of Physiology, Faculty of Medicine, “Iuliu Hațieganu” University of Medicine and Pharmacy, 400012 Cluj-Napoca, Romania; cristina.bidian@umfcluj.ro; 4 Department of Exact Sciences, Faculty of Horticulture, University of Agricultural Sciences and Veterinary Medicine Cluj-Napoca, 400372 Cluj-Napoca, Romania; popioana@usamvcluj.ro; 5 Department of Animal production and Food Safety, University of Agricultural Sciences and Veterinary Medicine Cluj-Napoca, 400372 Cluj-Napoca, Romania; adrian.macri@usamvcluj.ro

**Keywords:** 25 hydroxyvitamin D, overweight/obesity, type 2 diabetes mellitus, insulin resistance, systemic inflammation, oxidative stress

## Abstract

Vitamin D deficiency is highly prevalent in patients with overweight/obesity and type 2 diabetes (T2DM). Herein, we investigated the relationship between vitamin D status and overweight/obesity status, insulin resistance (IR), systemic inflammation as well as oxidative stress (OS). Anthropometric and laboratory assessments of 25-hydroxyvitamin D (25(OH)D) and glycemic, pro-inflammatory and OS biomarkers were performed in a sample of 47 patients with T2DM who were divided into categories based on overweight and degree of obesity. The main findings were: the overweight/obesity status correlated negatively with the degree of serum 25(OH)D deficiency (ρ = −0.27) with a trend towards statistical significance (*p* = 0.069); the homeostasis model assessment of insulin resistance (HOMA-IR) was significantly different (*p* = 0.024) in patients with 25(OH)D deficiency, as was total oxidant status (TOS) and oxidative stress index (OSI) in patients with severe serum 25(OH)D deficiency as compared to those with 25(OH)D over 20 ng/mL (TOS: *p* = 0.007, OSI: *p* = 0.008); and 25(OH)D had a negative indirect effect on TOS by body mass index (BMI), but BMI was not a significant mediator of the studied relationship. In a setting of overweight and increasing degree of obesity, patients with T2DM did not display decreasing values of 25(OH)D. Subjects with the lowest values of 25(OH)D presented the highest values of BMI. Patients with 25(OH)D deficiency were more insulin resistant and showed increased OS but no elevated systemic inflammation. The negative effect of 25(OH)D on TOS did not seem to involve BMI as a mediator.

## 1. Introduction

Emerging data have depicted vitamin D deficiency as a hallmark of obesity [1,2] Indeed, significantly lower serum vitamin D levels, as assessed by serum 25-hydroxyvitamin D (25(OH)D), have been reported in obese as compared to lean subjects [3,4]. Vitamin D shortage is also linked to high cardio-metabolic risk [5,6], which is a reflection of its extra-skeletal actions [1,7] such as modulating adipose tissue biology [8], glucose metabolism [9], inflammation as well as pro-oxidant/anti-oxidant balance [10], aside from its classic role in calcium hemostasis. On the other hand, obesity itself comprises several pathological features such as impaired insulin signaling, beta cell dysfunction, low-grade chronic inflammation and oxidative stress, which all result in insulin resistance (IR), and ultimately, in type 2 diabetes mellitus onset (T2DM) [11].

Overall, a negative correlation between 25(OH)D and body mass index (BMI) has been revealed in both patients with and without T2DM [12,13,14]. Yet, the relationship between vitamin D deficiency, obesity and T2DM as well as their underlying mechanisms, i.e., insulin-resistance (IR), low-grade systemic inflammation and oxidative stress is more complex and needs further investigation as studies report conflicting results [15]. Indeed, while some findings show a negative correlation between vitamin D and IR [16,17], others do not [18,19]. Also, some authors claim that vitamin D deficiency seems to be more related to glucose metabolism than to obesity [17] whereas others argue that the shortage is more associated with the degree of adiposity instead [20], suggesting that vitamin D does not play an important role in obesity-associated IR [21]. Furthermore, other authors [22] have suggested that the association between plasma 25(OH)D deficiency and IR in overweight and obese adults seems to be driven by central adiposity. In addition, a negative association between serum 25(OH)D and several biomarkers of systemic inflammation has been reported by several authors [23], regardless of the total quantity of fat mass, whereas others have showed no relationship between these parameters, which points to the lack of involvement of 25(OH)D in the pro-inflammatory milieu in obese individuals [24]. Finally, vitamin D shortage seems to be associated with depleted antioxidant status [25] and increased reactive oxygen species generation [26]. Given this gap in the understanding of the relationship between vitamin D shortage and obesity, T2DM and the underlying abnormalities, the objectives of our study were: (i) to investigate whether the degree of overweight/obesity is associated with various 25(OH)D deficiency levels; (ii) to analyze the relationship between 25(OH)D status and overweight/obesity, IR, systemic inflammation and oxidative stress; and (iii) to explore whether this putative relationship is mediated by BMI as an indicator of overweight/obesity status in a group of patients with T2DM.

## 2. Material and Methods

### 2.1. Study Design and Patients

This was an observational cross-sectional study with prospective data collection. We included 47 outpatients (27 female and 20 male) aged between 33 and 68 years old, with overweight or various degrees of obesity as described below, and with T2DM (between 12 years and newly diagnosed) that were enrolled from the Diabetes Ambulatory Care Unit of Medstar Medical Center, Cluj-Napoca, Romania in January and February 2018. The subjects were enrolled in the study in a consecutive manner. The exclusion criteria were supplementation with vitamin D, patients with T2DM treated with insulin, severe acute/chronic inflammatory diseases, renal/hepatic failure and neoplastic diseases.

The study was conducted according to the ethical principles of the Helsinki Declaration and was approved by the Ethics Committee of the “Iuliu Hațieganu” University of Medicine and Pharmacy (nr.458/20.12.2017). All patients gave their informed consent regarding their inclusion in this research project.

### 2.2. Anthropometric and Laboratory Assessment

Weight and height were determined by the usual means and BMI was calculated based on the formula, weight (kg)/squared height (m)^2^. Patients were classified as overweight (BMI 25–29.9 kg/m^2^), obesity class I (BMI 30–34.99 kg/m^2)^, class II (35–39.99 kg/m^2^) and class III/morbid obesity (BMI ≥ 40 kg/m^2^).

Blood samples were obtained after 8 h fasting, centrifuged and run immediately or stored at −80 °C until analyzed. Vitamin D was assessed as 25(OH)D and was considered deficient at serum levels of 10–20 ng/mL [27] and severely deficient at levels below 10 ng/mL [28,29]. We used the ELISA method (DiaMetra, Segrate, Italy) to determine the fasting levels of 25(OH)D as well as levels of interleukin 6 (IL-6) (Elabscience, Houston, TX, USA) and the chemiluminescent immunometric assay to evaluate the serum insulin levels (IMMULITE/IMMULITE 1000 insulin, Siemens, Munich, Germany). The intra- and inter-assay coefficients of variation for the tests were below 10%.

The quantification of serum nitric oxide (NO) concentration was performed through the evaluation of its final stable products (NO_x_), namely, nitrite (NO_2_^−^) and nitrate (NO_3_^−^) by using a colorimetric method [30]. NO_x_ was considered as both a marker of inflammation and nitro-oxidative stress as NO reacts with free radicals such as superoxide, which results in peroxynitrite formation [31]. Serum total oxidant status (TOS) and total antioxidant response (TAR) were determined by using colorimetric methods as well. TOS is mainly comprised of hydrogen peroxide and lipid hydroperoxide, while TAR refers mainly to free sulfhydryl groups of proteins, vitamin C, uric acid, bilirubin and vitamin E [32,33]. The methods are described in detail elsewhere [31]. The oxidative stress index (OSI) is the ratio TOS/TAR and this is an indicator of the degree of oxidative stress level: OSI (arbitrary unit) = TOS (μmol H_2_O_2_ equiv./L)/TAR (mmol Trolox equiv./L) [34].

Insulin resistance was evaluated by the homeostasis model assessment of insulin resistance (HOMA-IR) = (Fasting insulin (µUI/mL) × Fasting glucose (mg/dL))/405 [35].

### 2.3. Statistical Analysis

Quantitative continuous variables with a Gaussian distribution were summarized by the arithmetic mean (AM) and the standard deviation (SD). Continuous variables with a skewed distribution were described by the geometric mean (GM) and the geometric standard deviation (GSD, defined as the standard deviation of log-transformed data), with these two descriptive measures being used to describe the “center” and variability of log-transformed data. Quantitative continuous variables with severe departures from the Gaussian distribution were presented using the median (Me) and interquartile interval (IQR = (Q1; Q3), where Q1 = 25th percentile and Q3 = 75th percentile). The confidence intervals (95% CI) were also used to estimate the studied parameters in the population. The confidence intervals for the geometric mean were calculated in the traditional manner with the t-distribution, while confidence intervals for the median were calculated by the percentile method with 10,000 bootstrap samples.

The comparison of anthropometric and clinical variables with a Gaussian distribution among groups was performed using the one-way ANOVA test or Welch one-way test if the homoscedasticity was violated. The nonparametric Kruskal–Wallis test was used for data distributions that did not follow the normal probability law.

We used path analysis in order to test whether BMI (as an indicator of overweight/obesity status) is a mediator between 25(OH)D and oxidative stress. We tested the significance of indirect effects in a mediational path model. The fit of the conceptual model was evaluated by the Chi-square test and approximates fit indexes such as the comparative fit index (CFI) and Tucker–Lewis index (TLI). The approximate fit indices described the proportion of the improvement in the hypothetical model relative to a null model (a model assuming no correlation among the measured variables). A nonsignificant Chi-square test (*p* > 0.05), values of 0.90 or higher for CFI and TLI, a low value (<0.05) for the root mean square error of approximation (RMSEA) and a low value (<0.10) for the standardized root mean square residual (SRMR) were appropriate measures for the goodness-of-fit of the tested model [36]. The parameters of path analysis were estimated by the maximum likelihood estimation (MLE) method with bootstrapped standard errors. Since the variables used in the model were expressed in different measurement units, standardized path coefficients were also computed and represented in the path diagram in order to compare the size of the different effects.

All statistical analysis was performed in R v.4.0.3 (R Core Team (2020). R: A language and environment for statistical computing. R Foundation for Statistical Computing, Vienna, Austria. URL https://www.R-project.org/ (accessed on 30 October 2020)).

## 3. Results

### 3.1. General Characteristics Measured in Overweight/Obese Patients with T2DM

The demographic, anthropometric and clinical characteristics of the patients with T2DM stratified by overweight/obesity status are presented in Table 1. The overweight/obese groups were homogeneous by age with no significant difference in mean age (*p* = 0.888). We found no significant differences in the blood glucose (*p* > 0.05) or in the lipid profile and inflammatory status in the studied groups (Table 1). There was a significant difference in the geometric mean value of TOS (*p* = 0.006), the post-hoc analysis identified significant differences between class III/morbidly obese patients and overweight patients with T2DM (Tukey HSD test, adjusted *p*-value = 0.0032). The geometric TOS mean values were higher in class III/morbidly obese patients (GM = 35.97 μmoli H_2_O_2_/L, 95% CI for GM: 30.01–43.04) versus overweight patients (GM = 20.88, 95% CI for GM: 16.26–26.80). Also, the geometric OSI mean values were different in the tested groups with a tendency toward statistical significance (*p* = 0.073), being the highest in the morbidly obese patients (GM = 29.24, 95% CI for GM: 23.73–36.04) and the lowest in the overweight group (GM = 19.03, 95% CI for GM: 14.83–24.42).

### 3.2. Association between Serum Levels of 25(OH)D and Overweight/Degree of Obesity, Metabolic, Pro-Inflammatory and Oxidative Stress Biomarkers in Patients with T2DM

In our studied sample, 28 (59.57%) patients with T2DM had a severe deficiency of serum 25(OH)D (<10 ng/mL), 15 (31.91%) displayed serum 25(OH)D deficiency (10–20 ng/mL) levels whereas in 4 (8.5%) cases we identified values ≥ 20 ng/mL of 25(OH)D.

We found no significant differences in the distribution of serum 25(OH)D values (Kruskal–Wallis test, *p* = 0.669) in overweight and class I, II or III (morbid) obesity (Me (IQR): 10.90 ng/mL (4.65–17.20) for overweight, 10.20 (4.83–13.90) for patients with class I obesity, 7.30 ng/mL (4.70–13.85) for patients with class II obesity and 8.10 ng/mL (4.85–9.85) for patients with morbid obesity). However, we noticed a decreased monotonic relationship between the overweight/obese status and the degree of serum 25(OH)D deficiency (Spearman’s Rho coefficient, ρ = −0.27) with a trend towards statistical significance (*p* = 0.069).

There was a significant difference in the geometric mean value of HOMA-IR (*p* = 0.032, Table 2) and the post-hoc analysis identified a significant difference between patients with 25(OH)D deficiency versus patients with 25(OH)D over 20 ng/mL (Tukey HSD test, adjusted *p*-value = 0.024). Concerning the lipid profile, there was a significant difference in geometric mean value of triglycerides (*p* = 0.019), i.e., patients with severe serum 25(OH)D deficiency displayed a higher geometric mean value of triglyceride than patients with a level of 25(OH)D over 20 ng/mL (Tukey HSD test, adjusted *p*-value = 0.034, Table 2).

Regarding oxidative stress biomarkers, there were significant differences in the geometric mean values of TOS and OSI. The post-hoc analysis revealed significant differences between patients with a severe serum 25(OH)D deficiency and patients with 25(OH)D over 20 ng/mL (Tukey HSD test, adjusted *p*-value = 0.007 for TOS and adjusted *p*-value = 0.008 for OSI).

### 3.3. Path Analysis

The results of the correlation analysis are shown in Table 3. They reveal that there were significant linear correlations between 25(OH)D and TOS, and BMI and TOS (log-transformed variables).

The conceptual path model showed there was an adequate fit of the model to the data: Chi-square (χ^2^) statistics for null model = 23.51, *p*= 0.001, Chi-square (χ^2^) statistics for conceptual model = 0.06 df = 1, *p* = 0.802, RMSEA = 0.00, 90%CI: [0.00; 0.25], p_RMSEA ≤ 0.05_ = 0.813, SRMR = 0.012, CFI = 1.00, and TLI = 1.00.

**Figure 1:**
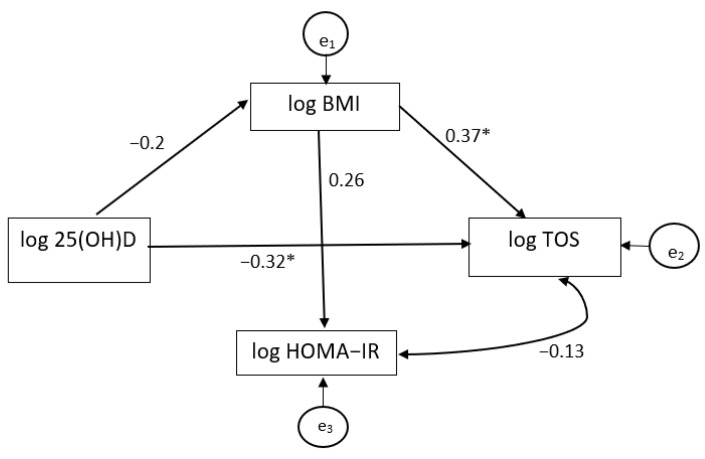
Path model for the effect of serum 25(OH)D concentrations on BMI and TOS (log-transformed variables) in overweight/obese patients with T2DM. Single-headed arrows indicate direct effect/relationships (effect size being represented by standardized path coefficients) while double-headed arrows indicate linear correlations; * *p* < 0.05: significant standardized path.

Table 4 summarizes the estimated direct and indirect effects of the exogenous variable (log 25OHVitD) on endogenous variables (log BMI, log TOS and log HOMA-IR). As shown in Table 4 and Figure 1, the log 25(OH)D had a significant negative direct effect on log TOS. A one ng/mL increase in log 25(OH)D resulted in a 0.20 μmol H_2_O_2_/L decrease in log TOS. Table 4 also shows the significant effect of log BMI on log TOS in which log TOS increased by 0.75 μmol H_2_O_2_/L for each one kg/m^2^ of log BMI increase.

In order to test and quantify the indirect effects of 25(OH)D on the TOS variable via BMI (log-transformed variables), we analyzed the standardized coefficient of the indirect effect calculated as a product of the direct effects. Although log 25(OH)D had a negative indirect effect on log TOS by log BMI, we did not find that BMI was a significant mediator of the relationship between 25(OH)D and TOS.

## 4. Discussion

The present study investigated the complex relationship between 25(OH)D status and overweight/obesity, IR, systemic inflammation and oxidative stress and revealed that: (1) Overweight and an increasing degree of obesity were not significantly associated with decreasing 25(OH)D levels. (2) A trend towards a significant negative correlation was observed between the overweight/obesity status and the degree of serum 25(OH)D deficiency. In addition, patients with the lowest values of 25(OH)D (<10 ng/mL) displayed the highest mean value of BMI (37.90 kg/m^2^). Patients with 25(OH)D deficiency were more insulin resistant and those with severe deficiency showed increased oxidative stress as compared to individuals with values over 20 ng/mL. However, interestingly, no differences were observed in terms of systemic inflammation in relation to the 25(OH)D status. Also, we found no significant correlations between 25(OH)D and IR and systemic inflammation while a negative correlation was observed between 25(OH)D and total oxidant status. (3) The negative effect of 25(OH)D on TOS was not mediated by BMI.

Vitamin D deficiency has emerged as a clinical syndrome induced by low serum levels of 25(OH)D, which is considered to be the most appropriate marker for the evaluation of vitamin D status as it comprises both intake and UV-sourced vitamin D [27,28]. The Endocrine Society Task Force defined vitamin D deficiency as values below a threshold of 20 ng/mL [27]. Also, a value below 10 ng/mL [29] is considered as a severe deficiency that leads to a high risk for chronic diseases [37]. Importantly, these values should be prevented and when they are not, proper supplementation is called for [38]. The 2011 report on dietary reference intakes for calcium and vitamin D state that serum 25(OH)D ≥ 20 ng/mL meets the requirements of 97.5% of the population [39].

Accumulating data have shown that vitamin D deficiency is a common feature of obesity [3,40,41]. Indeed, herein, we found that 59.57% of the overweight/obese patients with T2DM displayed a severe serum 25(OH) D (<10 ng/mL) deficiency, 31.91% showed a deficiency in the range between 10 ng/mL and 20 ng/mL whereas only 8.5% were identified with 25(OH)D values above 20 ng/mL. When looking at the overweight and increasing obesity categories, we did not notice decreasing levels of 25(OH)D levels as reported by Barrea et al. [42]. Also, although we did not find any correlation between 25(OH)D and BMI as reported in a recent review [14], a trend towards a significant negative correlation between the overweight/obesity status and the categories of serum 25(OH)D deficiency was revealed. Likewise, an inverse correlation between the different BMI classes and total 25(OH)D serum values was described by Vigna et al. [43]. Herein, the lack of statistical significance (r = −0.23, *p* = 0.127) when analyzing the correlation between 25(OH)D and BMI might be related to the small sample size but also to the means of the adiposity assessment. Indeed, some authors reported that it is the percentage/total body fat rather than BMI that is inversely associated with 25(OH)D [1,44,45]. Still, in our study, patients within the severe 25(OH)D deficiency category seem to have the highest BMI median level, i.e., 37.90 kg/m^2^. In line with our results, Piantanida et al. [46] reported that the highest percentage of patients in both categories of deficiency (below 10 and between 10–20 ng/mL) were morbidly obese (52% and 40%, respectively), and Bellia et al. [23] showed that when looking at severely obese individuals seeking bariatric surgery, patients in the lowest tertile of 25(OH)D were significantly more obese. The relationship between obesity and vitamin D deficiency is complex and remains a subject of debate. Patients with obesity are prone to develop a low vitamin D status as a consequence of lower dietary intake, reduced exposure to sunlight, decreased intestinal absorption, volumetric dilution as well as sequestration and accumulation in the large adipose tissue [47]. Ding et al. [48] showed that it is the adiposity markers (BMI and trunk fat percentage) that predict vitamin D deficiency and a reduced capacity to recover from this state. Moreover, De Pergola et al. [12] pointed out that adipose tissue accumulation per se is the main factor that drives, in an independent manner (independently of sex, body fat distribution, blood pressure and insulin, and metabolic parameters), the decrease in 25(OH)D values in patients with obesity. On the other hand, a reduced level of circulating 25(OH)D could by itself contribute to obesity development by inducing increased parathyroid hormone levels and the important inflow of calcium into adipocytes, resulting in lipogenesis and/or adipogenesis [40,41]. However, a bi-directional genetic study suggested that a higher BMI leads to lower 25(OH)D, whereas the effects of lower 25(OH)D on increasing BMI seem to be small [3]. Indeed, studies that analyzed the efficacy of vitamin D supplementation in obesity failed to report an influence on the adipose mass, leading to the assumption that it is obesity that induces a low vitamin D status rather than hypovitaminosis triggering obesity [49].

Several studies have reported the association of vitamin D deficiency not only with obesity, but with T2DM as well [50,51]. Indeed, a positive link between vitamin D shortage, obesity and T2DM has been reported, with a stronger negative association between 25(OH)D and BMI in patients with T2DM as compared to the non-diabetic group, and with the strongest correlation in the higher BMI quartile of the group of patients with T2DM [14]. Of note, many obese patients display glucose metabolism disturbances, which makes it difficult to analyze the relationship between vitamin D and T2DM because obesity acts as a confounding factor. Here, the lack of correlation between 25(OH)D and glucose metabolism markers and the presence of a trend towards a negative correlation between the categories of serum 25(OH)D deficiency and overweight/obesity status point to a link with overweight/obesity rather than with T2DM. Likewise, Horst et al. [21] reported no association between 25(OH)D deficiency and impaired glucose metabolism in morbidly obese women, but rather, with BMI and total body fat. On the other hand, Clemente-Postigo et al. [17] showed that 25(OH)D levels are diminished, but mostly in relation to carbohydrate metabolism, i.e., to T2DM rather than obesity. Similar to our results, the authors also found no differences in vitamin D values between BMI groups with the same glycemic status [17]. Indeed, the negative relationship between vitamin D and IR has been documented by some authors [16,17] but not by others [19,20]. Herein, we found no significant correlation between vitamin D and HOMA-IR, but we have to keep in mind that all the patients were overweight/obese with T2DM and most of them were medicated with metformin, which might limit the interpretation of the relationship with insulin sensitivity to some extent. Also, it may be that the absence of correlation between vitamin D and IR is a consequence of a blunting effect induced by obesity [52]. Still, the existence of an association between these two parameters emerges from the finding that patients with 25(OH)D deficiency were significantly more insulin-resistant as compared to those with 25(OH)D values over 20 ng/mL. Similar to our results, Bellia et al. [23] showed that severely obese patients eligible for bariatric surgery with the lowest 25(OH)D concentrations had a higher degree of IR. Vitamin D has been reported to be linked to glucose metabolism as a regulator of insulin secretion and as an enhancer of insulin sensitivity on target tissues, which gives room to the theory that hypovitaminosis D could be incriminated in the onset of IR, and ultimately, of T2DM [11,15]. However, Muscogiuri et al. [16] argue that there is no cause–effect relationship between vitamin D and insulin-sensitivity and that both low 25(OH)D value and IR seem to be driven by the increased body size, making it the main factor responsible for these changes in obese patients.

Both obesity and vitamin D shortage are associated with systemic inflammation and oxidative stress, which are interrelated and result in metabolic disturbances [53,54,55]. Previously, we have demonstrated that NO_x_ levels are increased in obese patients, and this is the outcome of the upregulation of inducible/inflammatory nitric oxide synthase (iNOS) induced by elevated inflammatory markers such as tumor necrosis factor alpha (TNF-α) [31]. Of note, NO_x_ is used for the indirect evaluation of NO production, and it is regarded as a marker of both inflammation and nitro-oxidative stress as NO reacts with free radicals such as superoxide, resulting in peroxynitrite formation [31]. Herein, we observed no significant differences in systemic inflammation with respect to 25(OH)D status and no correlations were found between 25(OH)D and pro-inflammatory markers (IL-6 and NO_x_), again, possibly as a consequence of the small number of patients included. Vitamin D has been reported to alleviate systemic inflammation in obese individuals by acting on both hypertrophic and dysfunctional adipocytes as well as on leucocyte infiltration [56,57]. More specifically, vitamin D is involved in down-regulation of NF-κB dependent transcription of pro-inflammatory genes that encode cytokines such as TNF-α, IL-6 and interleukin 8 (IL-8) [11,57]. Therefore, we would expect less systemic inflammation in patients with higher values of 25(OH)D; however, although we noticed a difference in median values, that is, subjects with 25(OH)D over 20 ng/mL displayed lower values of IL-6 (8.64 pg/mL) and those with severe deficiency showed higher IL-6 values (18.06 pg/mL), the differences in the distribution of IL-6 between the studied groups were not statistically significant. Still, the data regarding 25(OH)D and systemic inflammation are conflicting. Our results are in line with authors who reported no differences in IL-6 and high sensitivity C reactive protein (hs-CRP) with respect to different 25(OH)D status, i.e., inadequate, sufficient, and optimal, and no significant association between serum 25(OH)D and hs-PCR, IL-6 or TNF-α [58,59]. Indeed, Vilarrasa et al. [24] highlighted the lack of the involvement of 25(OH)D in the pro-inflammatory milieu in morbidly obese individuals. However, contrary to our results, Bellia et al. [23] provided evidence on the presence of a negative relationship between vitamin D levels and systemic inflammatory markers (also in a group of morbidly obese individuals) and argued that this association is independent of the total quantity of fat mass. In addition, 25(OH)D may be related to visceral adipose tissue-triggered inflammation, which in turn is involved in the onset of inflammatory status, even more markedly than BMI alone [23,60,61]. Altogether, the anti-inflammatory role of vitamin D is still debated and needs further investigation [59]. In addition, in a previous study, we reported an increased total oxidant status and a decreased total antioxidant response in morbidly obese patients as compared to normal weight individuals [31]. Here, we noted disparities between the categories of 25(OH)D deficiency in terms of oxidative stress status, namely, higher TOS and OSI in patients with severe serum 25(OH)D deficiency as compared to those displaying values over 20 ng/mL. Vitamin D has been reported to maintain balanced mitochondrial respiration and to prevent oxidative stress by reduction in the production of reactive oxygen species (ROS) and upregulation of antioxidants such as superoxide dismutase (SOD) and glutathione (GSH) [11,62]. Therefore, vitamin D shortage results in oxidative stress onset and here we showed that the total oxidant status was higher in patients with severe vitamin D deficiency as compared to those displaying higher levels of 25(OH)D; these findings are consistent with other reports [63]. A misbalance between pro-oxidant/anti-oxidant markers, namely, a decreased total antioxidant status in patients with lower vitamin D levels (but no increased biomarkers of oxidative stress across quartiles of plasma 25(OH)D) was also reported by Wang et al. [25]. In addition, here we identified a significant negative correlation between 25(OH)D and TOS and a significant negative direct effect on TOS, namely, an increase in 25(OH)D by one ng/mL resulted in a decrease in TOS by 0.20 μmoli H_2_O_2_/L. Further, given the data regarding both obesity- and vitamin D shortage-driven oxidative stress, we used path analysis in order to provide information on whether the relationship between 25(OH)D and TOS was mediated by BMI or not. Our results showed that BMI was not a mediator of the relationship between 25(OH)D and TOS, implying that the association between vitamin D deficiency and oxidative stress is most likely a direct one. However, Wang et al. [25] reported only a weak correlation between vitamin D shortage and the plasma non-enzymatic antioxidant capacity and no correlation with other markers of oxidative stress in healthy normal-weight adults. Importantly, these findings highlight the lack of a clear link between vitamin D status and oxidative stress in the absence of obesity and other non-communicable diseases.

We acknowledge that our findings are subject to some limitations. First, the size of the study group was small. Thus, caution is required in the interpretation of the results of the path analysis, and future studies based on larger samples should retest the proposed path model. Second, the cross-sectional design of the study precluded us from analyzing the cause–effect relationship between the studied parameters. Third, we did not collect information on dietary habits, and therefore on vitamin D intake from food as well as on sunshine exposure. Notably, the data collection was realized in January and February. Therefore, the relationship between vitamin D and the studied parameters should be reanalyzed in future studies in order to determine whether they are influenced by seasonal variations. Finally, the IR was assessed by HOMA-IR, not by the gold standard euglycemic–hyperinsulinemic clamp and the total antioxidant response did not include antioxidant enzymes such as superoxide dismutase, glutathione peroxidase and catalase.

## 5. Conclusions

In a setting of overweight and increasing degree of obesity, patients with T2DM did not display decreasing values of vitamin D. However, individuals with the lowest values of vitamin D had the highest values of BMI. In addition, the degree of vitamin D deficiency tended to be negatively associated with the overweight/obesity status. Patients with vitamin D deficiency were more insulin resistant and showed increased oxidative stress (those with severe deficiency) but no elevated systemic inflammation as compared to individuals with vitamin D values over 20 ng/mL. Finally, the negative effect of vitamin D deficiency on total oxidant status did not seem to involve BMI as a mediator.

## Figures and Tables

**Table 1:** Main demographic, anthropometric and laboratory characteristics according to overweight/obesity status (*n* = 47, 27 female, 20 male).

		Overweight/Obesity Status			
	Overweight (*n*_1_ = 7)	Class I Obesity (*n*_2_ = 10)	Class II Obesity (*n*_3_ = 15)	Class III/Morbid Obesity (*n*_4_ = 15)	*p*-Value
Age (years) ^(a)^	57.86 ± 2.04	57.80 ± 4.13	57.00 ± 9.09	55.80 ± 7.97	0.888
BMI (kg/m^2^) ^(b)^	28.66 (27.06–28.99)	32.31 (31.24–33.46)	35.94 (35.56–36.62)	44.70 (42.43–45.86)	<0.0001 ***
Blood glucose (mg/dL) ^(c)^	133.77 ± 1.19	127.01 ± 1.15	128.87 ± 1.19	134.53 ± 1.21	0.827
Insulin (μIU/mL) ^(c)^	10.14 ± 2.10	20.23 ± 2.21	19.96 ± 1.59	19.34 ± 1.93	0.110
HOMA-IR ^(c)^	3.35 ± 1.97	6.35 ± 2.39	6.35 ± 1.63	6.42 ± 2.21	0.195
Total cholesterol (mg/dL) ^(c)^	148.56 ± 1.33	186.60 ± 1.26	175.02 ± 1.29	174.83 ± 1.37	0.411
Tryglicerides (mg/dL) ^(c)^	115.57 ± 1.52	120.19 ± 1.51	121.92 ± 1.73	142.78 ± 1.36	0.637
IL-6 (pg/mL) ^(a)^	25.00 ± 21.41	14.58 ± 8.77	21.04 ± 15.49	16.26 ± 8.98	0.468
NO_x_ (μmol/L) ^(a)^	50.09 ± 24.96	64.78 ± 18.73	62.96 ± 20.96	65.57 ± 12.55	0.323
TOS (μmol H_2_O_2_ equiv./L) ^(c)^	20.88 ± 1.31	27.31 ± 1.50	28.82 ± 1.31	35.97 ± 1.38	0.006 **
TAR (mmol Trolox equiv./L) ^(a)^	1.094 ± 0.001	1.093 ± 0.002	1.094 ± 0.002	1.094 ± 0.001	0.758
OSI ^(c)^	19.03 ± 1.31	23.58 ± 1.58	26.02 ± 1.32	29.24 ± 1.46	0.073

^(a)^ arithmetic mean (AM) ± standard deviation (SD); ^(b)^ median (IQR: lower quartile-upper quartile); ^(c)^ geometric mean (GM) ± geometric standard deviation (GSD); *p*-values obtained from one-way ANOVA test, Welch one-way test or Kruskal–Wallis test; for the variables described by geometric mean and geometric standard deviation, statistical tests were applied for log-transformed data; ** statistically very significant result: *p* < 0.01; *** statistically extremely significant result: *p* < 0.001; BMI: body mass index; HOMA-IR: homeostasis model assessment of insulin resistance; IL-6: interleukin 6; NO_x_: nitrites/nitrates; TOS: total oxidant status; TAR: total antioxidant response; OSI: oxidative stress index.

**Table 2:** Overweight/obesity status, metabolic, pro-inflammatory and oxidative stress biomarkers by categories of serum 25(OH)D.

Serum 25(OH)D (ng/mL)	<10 ng/mL (*n*_1_ = 28)	10–20 ng/mL (*n*_2_ = 15)	≥20 ng/mL (*n*_3_ = 4)	*p*-Value
BMI (kg/m^2^) GM (95% CI)	37.90 (35.44–40.53)	34.85 (31.90–38.07)	30.59 (23.04–40.60)	0.043 *
HOMA-IR, GM (95% CI)	5.73 (4.40–7.46)	7.35 (4.91–10.99)	2.56 (1.16–5.66)	0.032 *
Total cholesterol (mg/dL), GM (95% CI)	185.31 (168.23–204.13)	160.44 (134.35–191.61)	142.63 (132.48–153.57)	0.087
Tryglicerides (mg/dL), GM (95% CI)	143.75 (125.87–164.17)	112.50 (84.33–150.07)	82.68 (63.61–107.46)	0.019 *
IL-6 (pg/mL), ME (95% CI)	18.06 (14.19–24.21)	14.81 (5.96–18.18)	8.64 (1.02–23.82)	0.228
NO_x_ (μmol/L), AM (95% CI)	65.60 (58.59–72.61)	59.33 (47.74–70.91)	49.90 (28.98–70.82)	0.235
TOS (μmol H_2_O_2_ equiv./L), GM (95% CI)	32.33 (28.30–36.93)	27.10 (22.62–32.48)	18.54 (15.66–21.96)	0.007 **
TAR (mmol Trolox equiv./L), AM (95% CI)	1.0932 (1.0909–1.0956)	1.0936 (1.0925–1.0947)	1.0936 (1.0931–1.0941)	0.908
OSI, GM (95% CI)	28.49 (24.77–32.77)	22.69 (19.09–26.97)	16.14 (12.04–21.64)	0.005 **

GM = geometric mean; AM = arithmetic mean; ME = median point estimation; one-way ANOVA or Kruskal–Wallis test; 95% confidence intervals (CI) for the geometric mean are calculated in the traditional manner with the t-distribution; 95% confidence intervals (CI) for the median are calculated by the percentile method with 10,000 bootstrap samples; * statistically significant result: *p* < 0.05; ** statistically very significant result: *p* < 0.01; 25(OH)D: serum 25-hydroxyvitamin D; BMI: body mass index; HOMA-IR: homeostasis model assessment of insulin resistance; IL-6: interleukin 6; NO_x_: nitrites/nitrates; TOS: total oxidant status; TAR: total antioxidant response; OSI: oxidative stress index.

**Table 3:** Matrix of the Pearson correlation coefficient (*p*-values) among studied variables.

	Log 25(OH)D	Log BMI	Log TOS	Log HOMA-IR
Log25(OH)D	*1*			
logBMI	−0.23 (0.127)	*1*		
logTOS	**−0.41 (0.004)**	**0.45 (0.0016)**	*1*	
LogHOMA-IR	−0.02 (0.873)	*0.26 (0.078)*	0.00 (0.873)	*1*

Bold denotes significant correlations: *p* < 0.05; italics denote the correlations with marginal statistical significance: 0.05 ≤ *p* < 0.10.

**Table 4:** Path coefficients of the conceptual model.

	Unstandardized Path Coefficient $(\hat{b})$	SE	Z-Statistics	p (Z > |z|)	Percentile 95% CI
Direct effect					
log 25(OH)D → log BMI	−0.07	0.04	−1.59	0.113	(−0.15; 0.02)
log 25(OH)D → log TOS	−0.20	0.08	−2.46	0.014 *	(−0.36; −0.03)
log BMI → log TOS	0.75	0.27	2.76	0.006 *	(0.21; 1.29)
log BMI → log HOMA-IR	*1.05*	*0.56*	*1.87*	*0.061*	(*−0.10*; *2.14*)
Indirect effect					
log 25(OH)D → log BMI → log TOS	−0.05	0.04	−1.41	0.159	(−0.13; 0.02)

→ = effect; SE = bootstrapped standard errors; Percentile CI = percentile bootstrap confidence interval for point estimate of unstandardized path coefficients; 10,000 bootstrap samples, the point estimate was considered significant when the confidence interval did not contain zero; * statistically significant result: *p* < 0.05; the results with a marginal significance were marked in italics.

## Data Availability

The raw data involved in this study can be obtained upon reasonable request addressed to Adriana Florinela Cătoi (adriana.catoi@umfcluj.ro).
